# Advances in Cancer Research: Current and Future Diagnostic and Therapeutic Strategies

**DOI:** 10.3390/bios14020100

**Published:** 2024-02-16

**Authors:** Xiaohui Liu, Hui Jiang, Xuemei Wang

**Affiliations:** State Key Laboratory of Digital Medical Engineering, School of Biological Science and Medical Engineering, Southeast University, Nanjing 210096, China; sungi@seu.edu.cn

**Keywords:** cancers of unknown primary (CUP), diagnostics, targeted therapy, bio-nanotechnology, artificial intelligence (AI), microfluidic

## Abstract

Cancers of unknown primary (CUP) exhibit significant cellular heterogeneity and malignancy, which poses significant challenges for diagnosis and treatment. Recent years have seen deeper insights into the imaging, pathology, and genetic characteristics of CUP, driven by interdisciplinary collaboration and the evolution of diagnostic and therapeutic strategies. However, due to their insidious onset, lack of evidence-based medicine, and limited clinical understanding, diagnosing and treating CUP remain a significant challenge. To inspire more creative and fantastic research, herein, we report and highlight recent advances in the diagnosis and therapeutic strategies of CUP. Specifically, we discuss advanced diagnostic technologies, including 12-deoxy-2-[fluorine-18]fluoro-D-glucose integrated with computed tomography (18F-FDG PET/CT) or ^68^Ga-FAPI (fibroblast activation protein inhibitor) PET/CT, liquid biopsy, molecular diagnostics, self-assembling nanotechnology, and artificial intelligence (AI). In particular, the discussion will extend to the effective treatment techniques currently available, such as targeted therapies, immunotherapies, and bio-nanotechnology-based therapeutics. Finally, a novel perspective on the challenges and directions for future CUP diagnostic and therapeutic strategies is discussed.

## 1. Introduction

**Figure 1:**
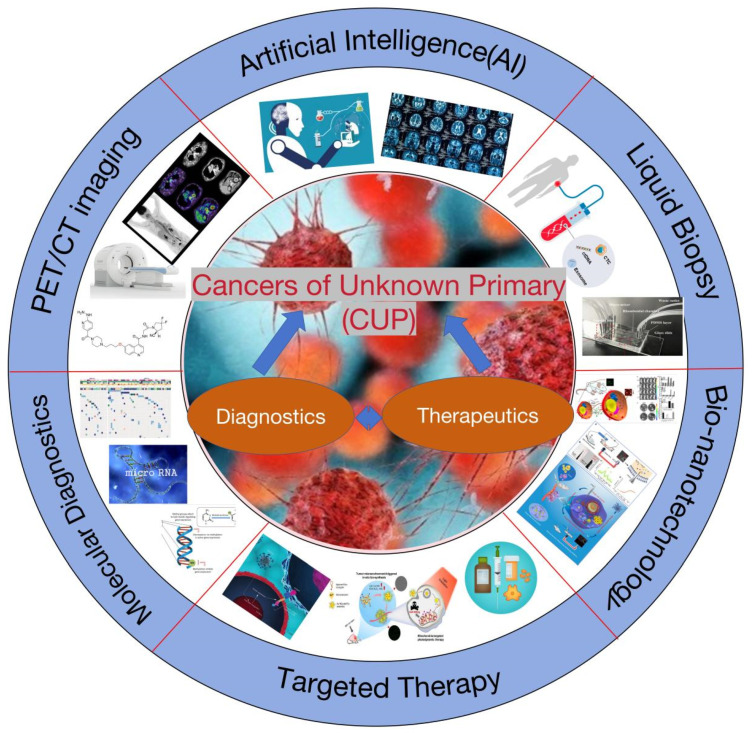
Diagnostic and therapeutic strategies for cancers of unknown primary (CUP).

In the oncological diagnostic and treatment system, identifying the primary site is fundamental for standardized treatment. Yet, 3–5% of cancers remain with undetermined primary sites after pathological diagnosis, classified as CUP [1]. CUP’s incidence ranges from 6 to 16 per 100,000, accounting for 2.3% to 7.8% of all malignant tumors, and it ranks fourth in mortality. The discovery of the primary site poses a significant challenge due to a lack of effective detection methods [2]. As a result, 20–50% of patients do not have an identifiable primary site, and most CUP primary lesions found during autopsies are less than 1 cm, undetectable by current technologies [3,4,5]. Hence, summarizing and discussing the research progress in early detection, precise diagnosis, and targeted treatment strategies for CUP are of paramount importance (Figure 1).

Another major challenge is the treatment and prognosis of CUP patients, with median survival rates reported between 2 and 12 months [6]. Treatment primarily involves empirical chemotherapy (e.g., taxane and platinum-based regimens), resulting in generally poor prognoses [7]. Thus, it is urgent to identify the primary site and understand the cancer’s origin and tissue type, which enables treatment according to the type of primary cancer, aiding clinicians in selecting the most appropriate treatment plan [8]. Moreover, most patients diagnosed with CUP present with metastases, commonly in the lungs, liver, bones, and lymph nodes [9]. The mechanisms behind cancer spreading or metastasizing from such small sites remain unclear, making research into new therapeutic techniques targeting CUP crucial for understanding their pathogenesis [10,11,12]. This review summarizes and discusses recent international research progress on CUP, including 18F-FDG PET/CT whole-body imaging, liquid biopsy, molecular diagnostics, nanoprobes, particularly in vivo self-assembling probes for diagnostics and therapy, and AI-based diagnostic technologies. Additionally, it delves into molecular targeted therapies, immunotherapies, and biotechnological treatment strategies for CUP.

## 2. Diagnostic Techniques for Cancers of Unknown Primary (CUP)

Clinical imaging tests for CUP typically include X-ray, CT, MRI, and PET/CT techniques. These tests can aid in a more accurate diagnosis of the tumor by determining its location, size, shape, and association with surrounding tissues. It is essential that the primary site of a tumor be detected early and diagnosed accurately. Therefore, identifying the primary site and accurately understanding the tumor’s origin and tissue type are vital for guiding appropriate treatment strategies [13]. The difficulty in the diagnosis of CUP is a result of the following factors: primary tumors may be too small, slow-growing, and undetectable by current imaging or other diagnostic techniques; metastasized tumor cells might have altered morphologies, thus not resembling the original primary site; primary lesions could be eliminated by the body’s immune system (no longer existing primary sites); and primary tumors might have been inadvertently removed or destroyed during surgery or treatment. Studies have shown that in highly differentiated cancers, oncogenes in metastatic cells can be used to trace the tissue of origin. Therefore, immunohistochemistry (IHC) plays a critical role in the assessment of metastatic sites [14]. However, due to the high heterogeneity of CUP tumors, conventional pathological diagnostic techniques have limitations, such as insufficient tumor sampling, specimen fixation affecting tumor antigenicity, observer subjectivity, and numerous clinical interfering factors, which do not entirely meet clinical needs. To overcome these challenges, researchers have explored new technologies, such as ^18^F-FDG PET/CT, liquid biopsy, molecular diagnostics, in vivo self-assembling probes, and AI-based identification for multimodal imaging and spectroscopic analysis of CUP, providing evidence-based support for diagnosis at multiple levels and scales [15]. This section will focus on discussing the application and research progress of these technologies in diagnosing CUP, while also highlighting their potential shortcomings and limitations.

### 2.1. PET/CT Imaging

**Figure 2:**
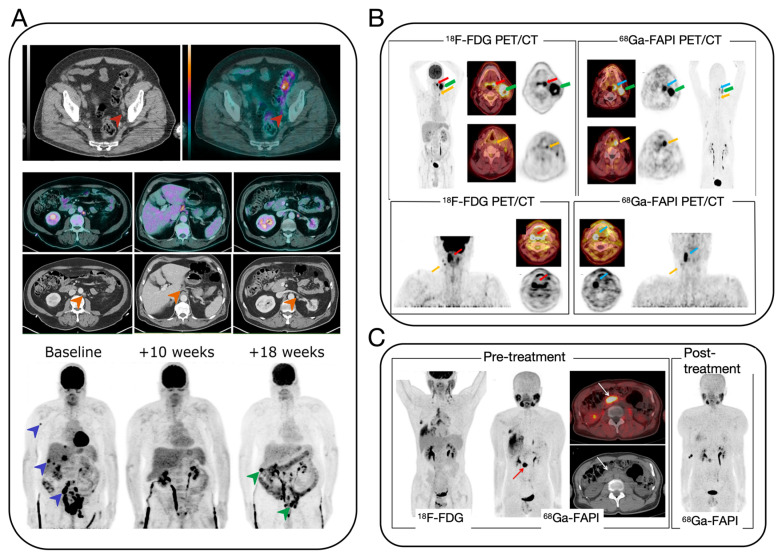
(**A**) ^18^F-FDG PET/CT images of three patients at different time points in the course of MIBC [19]. (**B**) PET/CT scans with the FDG and FAPI tracer of patient with palatine tonsil carcinoma and simultaneous hypopharyngeal carcinoma [23]. (**C**) Pre- and post-treatment PET/CT images of a 58-year-old man with IgG4-related disease [24].

The early and accurate identification of the primary site of CUP is critical for patient diagnosis and treatment. Computed tomography (CT) and magnetic resonance imaging (MRI) are the most widely used techniques in clinical applications [16]. CT imaging is extensively utilized to locate and stage the primary site in CUP patients, particularly sensitive for primary sites in the lungs, pancreas, or kidneys and advantageous for identifying metastatic sites in the liver, lungs, and bones. However, it may miss smaller lesions and/or unenhanced lesions with no abnormal morphological or vascular changes. PET/CT imaging, a non-invasive method, integrates molecular-level metabolic imaging with anatomical changes, simultaneously providing precise localization and characterization of lesions, thereby complementing each other’s strengths. ^18^F-FDG, a glucose analog, is phosphorylated by hexokinase after intravenous injection but, unlike normal glucose, does not participate in further metabolism and remains within cells. There is a significant increase in glucose metabolism in most malignant tumors [17]. Studies show that the concentration of ^18^F-FDG can effectively distinguish malignant from benign lesions, significantly improving the accuracy of diagnosis [18]. ^18^F-FDG PET/CT imaging has shown high diagnostic value in identifying primary tumor lesions, with studies indicating a reduction in CUP cases among head and neck cancers from 2–9% to 1–2% following the adoption of PET/CT (Figure 2A) [19]. Furthermore, PET/CT imaging can simultaneously detect other metastatic sites and their extent, significantly impacting tumor staging, restaging, and treatment. PET/CT plays a crucial role in CUP diagnosis. However, there are some problems in clinical application: Firstly, PET/CT examinations require the use of radioisotopes, which expose patients to radiation. Although the half-life of these isotopes is very short, high doses of radiation may still increase the risk of cancer. Secondly, PET/CT examinations can be expensive and may not be readily available to all patients. PET/CT equipment is expensive and requires complex maintenance, leading to increased CUP screening costs. Furthermore, it may be challenging to access this equipment in certain regions, resulting in CUP patients being referred or placed on waiting lists. Additionally, contrast allergy is a potential concern. During PET/CT examinations, some CUP patients may require the use of iodine-containing contrast agents, which can cause allergic reactions. Although this is a rare occurrence, it is still important to take precautions. Also, interpreting PET/CT images can be challenging due to their complexity and may require the expertise of professional radiologists. However, even experienced doctors may have difficulty interpreting medical imaging due to the rapid development and upgrading of technology. Lastly, PET/CT alone may not accurately detect lesions smaller than a certain size, which can impact early disease diagnosis and treatment. Despite its limitations, PET/CT remains an important tool in CUP diagnosis. It is crucial to carefully consider the benefits and drawbacks of PET/CT.

There are other methods available for CUP diagnosis and monitoring, such as nuclear medicine-based molecular imaging modalities like PET/CT and SPECT/CT, as well as anatomical modalities like CT and MRI. There are several radiotracers in research in addition to the gold standard, i.e., 18F-FDG and [^68^Ga]Ga-FAPI-4 PET/CT. Studies have found that despite the advancements in ^18^F-FDG PET/CT improving detection rates in CUP patients, it struggles to identify very small or low-activity lesions [19]. Although various tumor markers in the blood are related to the type of primary tumor, their specificity and sensitivity are not very high. Research has shown that to enhance the accuracy of detecting primary tumors, a novel PET tracer, [^68^Ga]Ga-FAPI-4, targeting the expression of fibroblast activation protein (FAP) has been utilized for enhancing the specificity of cancer imaging [20,21,22,23,24]. Experimental comparisons indicate that [^68^Ga]Ga-FAPI-4 can be a more specific new target tracer for cancer imaging [23,24] (Figure 2B,C). Thus, [^68^Ga]Ga-FAPI-4 represents a significant advancement in tracers following 18F-FDG. Future research and development of new tracers will be a key direction to improve the accuracy, resolution, and safety of CUP diagnosis.

### 2.2. Molecular Diagnostics

**Figure 3:**
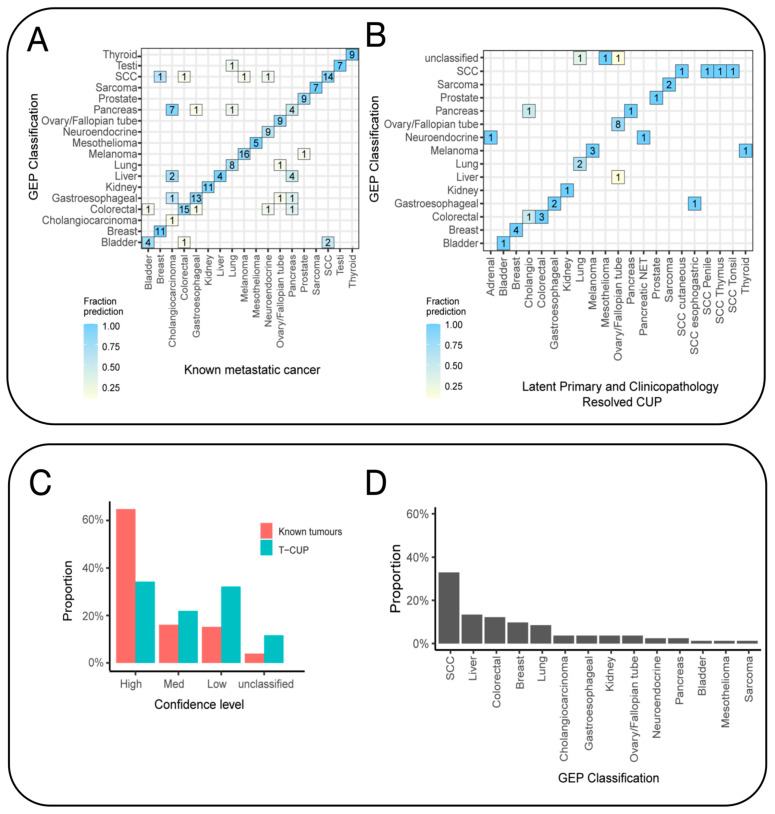
Gene expression profiling (GEP) tissue-of-origin classification of known metastatic cancers and SUPER cancer of unknown primary (CUP) tumors. (**A**) The confusion matrix shows that the tissue-of-origin prediction was concordant with the known cancer type. (**B**) The GEP classifier was also tested on clinicopathology-resolved CUPs, and the results showed concordance between the likely tissue of origin and the predicted cancer type. The analysis excluded latent primary and clinicopathology-resolved CUPs that represent cancer types not included in the classifier model. (**C**) The fraction of cases within the confidence probability score grouping contrasts the classification of clinicopathology-unresolved CUPs and clinicopathology-resolved CUPs combined with known metastatic tumors. Cases are classified as unclassified if the score is less than 0.5, low if it is between 0.5 and 0.7, medium confidence if it is between 0.8 and 0.9, and high confidence if it is 1. (**D**) The GEP can predict the cancer class of all clinicopathology-unresolved CUPs with high–medium confidence [29].

Patients with cancer of unknown primary often undergo extensive diagnostic tests, including pathology, radiology, endoscopic, and laboratory examinations, to determine the primary site of the cancer. Patients with this condition are typically treated with empirical combination chemotherapy and have a poor prognosis. Recent studies have suggested the use of genomics and transcriptomics to identify the primary origin. Based on the hypothesis that metastatic tumors retain similar molecular spectra to their primary tumors, researchers have developed various molecular diagnostic methods, including gene expression profiling (GEP), gene mutation spectra, and DNA methylation [25,26,27]. Among these, GEP is a crucial technique for tracing CUP origins [28]. The gene expression profile of a metastatic tumor differs from that of the tissue at the metastatic site, but it is more similar to the expression profile of the primary site. This suggests that tumors maintain the gene expression traits of their tissue origin during both development and metastasis. Using this principle, researchers have developed GEP methods to identify the primary site of tumors, especially in poorly differentiated/undifferentiated cancers [29] (Figure 3). Due to the difficulty in determining the origin of most CUP cases, the accuracy of GEP in cryptic primary tumors remains uncertain. Beyond GEP, Laprovitera and colleagues used digital PCR to assess the expression of 89 microRNAs in 159 FFPE samples, including primary tumors from 17 types (reference set) and metastatic tumors of known or unknown origin (test set), achieving 60% accuracy in 81% of cases [30]. Additionally, a set of 13 microRNAs was shown to have prognostic value, correlating with the overall survival rate of CUP patients.

Several commercial molecular diagnostic platforms have been established worldwide in recent years for diagnosing the tissue of origin in CUP patients. Rosetta Genomics Laboratory in Israel accurately identified the origin of FFPE tumor samples by analyzing the expression levels of 48 microRNAs [31]. This process results in the detection of 42 types of tumors with an accuracy rate of 85%. Biotheranostics Laboratory in the United States utilizes real-time reverse-transcription polymerase chain reaction (RT-PCR) to detect the expression of 92 genes in patient tumors, classifying them by matching gene expression patterns with a database of over 2000 known tumor types and subtypes, differentiating 50 different tumor types and subtypes, covering over 95% of solid tumors, with an accuracy rate of 85% [32]. Currently, artificial intelligence (AI) and bioinformatics-based multi-omics analysis technologies are also increasingly used in identifying the origin of CUP, emerging as high-performance, low-cost methods for cancer tracing, and they are expected to further improve the accuracy of identification and the efficacy of guided therapy [33].

#### 2.2.1. Liquid Biopsy

**Figure 4:**
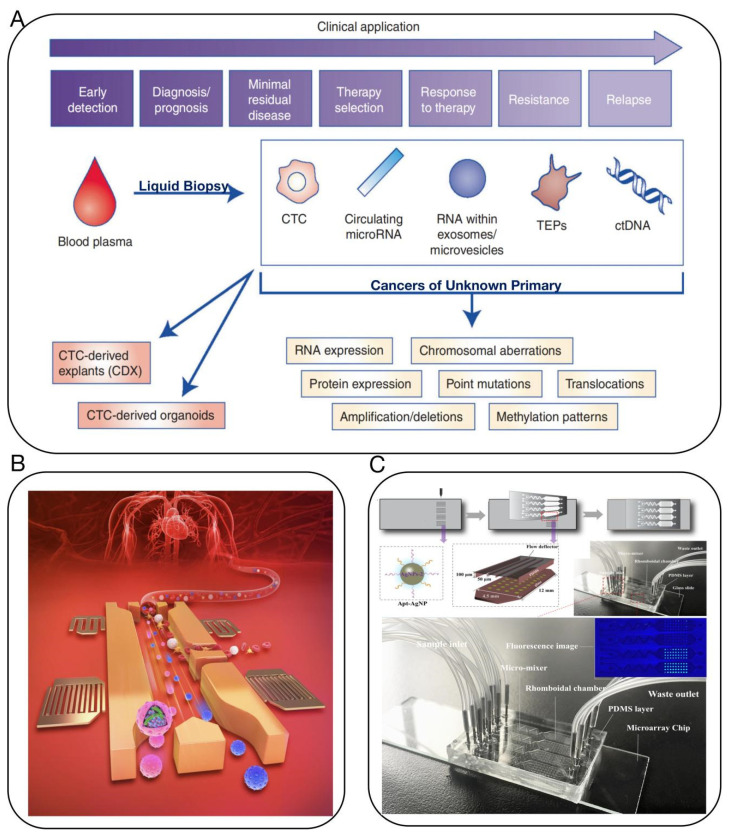
(**A**) Potential clinical and research applications of liquid biopsies for the management of CUP. Clinical and research applications of liquid biopsies are wide-reaching, from early detection and diagnosis to monitoring response to therapy and earlier detection of disease relapse [36]. (**B**) Microfluidic device uses acoustics to quickly analyze blood for signatures of cancer and other diseases [37]. (**C**) Multivalent aptasensor array and silver-aggregated amplification for multiplex detection in microfluidic devices [38].

Compared to traditional cancer screening techniques, liquid biopsy plays a significant role in early tumor diagnosis, precision treatment, progression and metastasis, heterogeneity and resistance, and prognostic assessment [34,35,36,37,38]. It has been recognized by the Massachusetts Institute of Technology (MIT) Technology Review as “one of the top ten breakthrough technologies of 2015” and has the potential to revolutionize the precise diagnosis and treatment of CUP [39,40]. Liquid biopsy, a novel non-invasive method, allows for the examination of non-solid tissue samples from CUP. During apoptosis or metastasis, CUP cells release small molecular substances such as DNA/RNA into the bodily fluids, which can be detected and sequenced for genomic analysis, aiding in the identification of the original tumor site, assisting in diagnostic typing, and adjusting treatment plans [36,41] (Figure 4A). In comparison to traditional tissue testing, liquid biopsy offers several advantages: it is non-invasive, well-tolerated by patients, allows for dynamic monitoring, and overcomes tumor heterogeneity. Commonly used techniques for the detection of circulating tumor DNA (ctDNA) include the Amplification Refractory Mutation System (ARMS), next-gen sequencing (NGS), digital PCR (dPCR), and nucleic acid mass spectrometry [42]. These diagnostic tools are widely utilized for the detection of cancers of unknown primary (CUP) [43]. Researchers from Johns Hopkins University and the Mayo Clinic have developed a new liquid biopsy technology, combining protein biomarkers with cell-free DNA (cfDNA) at the gene level, targeting 9 protein markers and 61 regions across 16 genes [44]. This test was performed in 1005 patients diagnosed with non-metastatic cancers, including ovarian, liver, stomach, pancreas, esophagus, colon, lung, and breast cancer. An average of 70% positivity was observed across these eight cancer types, with detection sensitivity ranging from 69 to 98% and specificity over 99%.

However, ctDNA only constitutes 0.1–5% of cfDNA in actual samples, demanding detection techniques with sufficiently low detection limits and high sensitivity to meet liquid biopsy requirements [45,46]. Moreover, strong background interference in bodily fluids and the minute amount of information available in liquid biopsies can lead to biases in analysis. Therefore, liquid biopsy demands high-quality control throughout the analysis process, minimizing the fragmentation and degradation of ctDNA to avoid false-negative results. The integration of microfluidic chips with liquid biopsy technology can address these issues to some extent and has been increasingly used in the sample preparation, separation, and detection of clinical samples [35,37,38,47,48] (Figure 4B,C).

The challenge with liquid biopsy is clearly the lack of sensitivity of current techniques to low-shedding tumors, as all tumor tissues theoretically shed their DNA, RNA, or other material into the bloodstream. However, there are some tumors, such as glioblastomas, that rarely shed their genetic material, etc., into the bloodstream due to anatomical location or histological features. The same problem arises in the sensitivity of detection at different stages of the disease, where we have made considerable progress in detecting tumors at advanced stages of the disease, such as stage Ⅳ in tumors that have developed metastases, but there are limitations at stage Ⅲ, Ⅱ, or Ⅰ. In addition, NGS sequencing of liquid biopsies is expensive and often provided by centralized laboratories, so “decentralisation” would be more conducive to clinical dissemination and application.

#### 2.2.2. Artificial Intelligence (AI)

**Figure 5:**
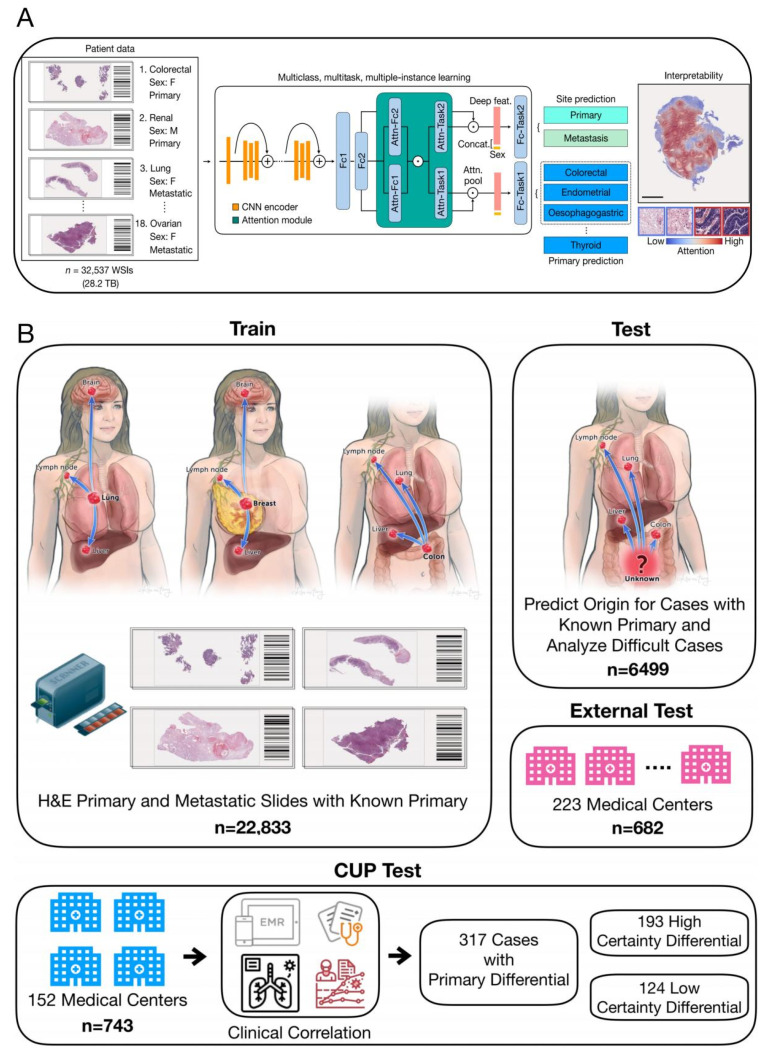
(**A**) TOAD workflow: The model was initially trained and tested on tumors with confirmed primary origins. The model was assessed on progressively challenging cases of metastatic tumors. Finally, it was evaluated to determine its ability to provide meaningful predictions for the origins of cancers that cannot be easily diagnosed by human experts using H&E histology alone. (**B**) The TOAD workflow involves using digitized high-resolution histology slides as input into the main network. An attention-based multiple-instance learning algorithm is used to rank all tissue regions in the slide based on their feature vectors and aggregate their information across the whole slide. This is achieved by assigning greater weights to regions perceived to have high diagnostic relevance. To further guide classification, the sex of the patient can be added as a covariate to the aggregated histology features. TOAD uses a multi-branched network architecture and a multitask objective to predict both the tumor origin and whether the cancer is primary or metastatic. The network’s attention scores for each region can also be used to interpret the model’s prediction. Scale bar, 0.5 mm. Attn, attention; Concat, concatenation; Fc1, Fc2, fully connected layers; feat, features; F, female; M, male [49].

Artificial intelligence (AI), as an emerging disruptive technology, is gradually unleashing the immense energy of the technological revolution and industrial transformation, continually expanding its application domains [33]. Recently, a team from Harvard Medical School, led by Faisal Mahmood, published a research paper in *Nature*, titled “AI-based pathology predicts origins for cancers of unknown primary” [49] (Figure 5). This study introduced a deep learning-based algorithm, tumor origin assessment via deep learning (TOAD), utilizing conventionally obtained histological slides to provide a differential diagnosis for the origins of primary tumors. Deep learning serves as an auxiliary tool, offering differential diagnosis for complex cases of metastatic tumors and CUP, and it can be used in conjunction with or as an alternative to auxiliary examinations and comprehensive diagnostic tests, reducing the incidence of CUP. The research team trained the AI system with billion-pixel pathology whole-slide images from over 22,000 cancer cases, then tested it on approximately 6500 cases with known primary cancers and analyzed increasingly complex cases of metastatic cancer to establish the AI model’s analytical capabilities for CUP. For tumors with known primary sites, the AI model achieved a prediction accuracy of 83%, with a top-3 prediction accuracy of 96%. The model was then tested on 317 cases of CUP, showing a diagnostic concordance rate of 63% with pathologists and a top-3 diagnostic concordance rate of 82%. This AI model’s performance is roughly equivalent to several recent studies using genomic data to predict tumor origins. However, the application of intelligent medical imaging is limited to a single disease and requires separate algorithm training and design development for different diseases. How to quickly develop new products for different types of diseases, different modality data, or multi-modality fusion is an industry challenge. In addition, diagnostic accuracy for CUP and other diagnostic accuracy are the core performance of intelligent medical imaging products. Currently, most of the performance parameters released by intelligent medical imaging products come from limited datasets and laboratory conditions, and due to factors such as the number of datasets and insufficient representativeness, the actual detection performance of the products is not good enough in highly complex clinical applications; thus, the robustness of the products needs to be improved. Currently, deep learning algorithms commonly applied in the field of artificial intelligence need to construct multi-hidden-layer neural networks. The prediction process is a computational process under the corresponding parameters, and this process is opaque, so the prediction results are not interpretable. Therefore, the application of AI-based diagnostic methods in the clinic still has a long way to go.

**Figure 6:**
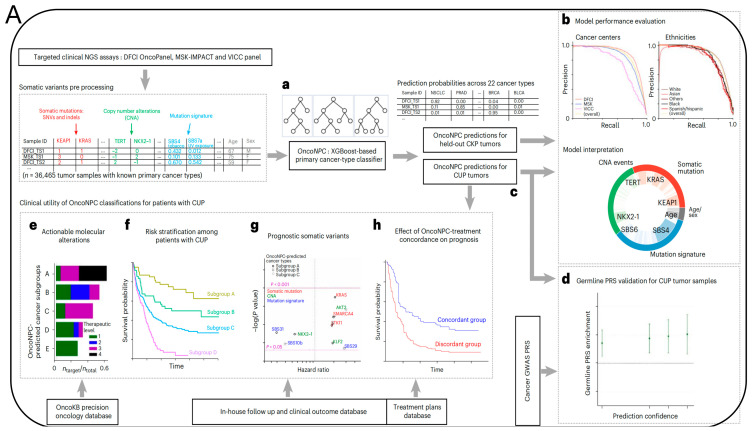

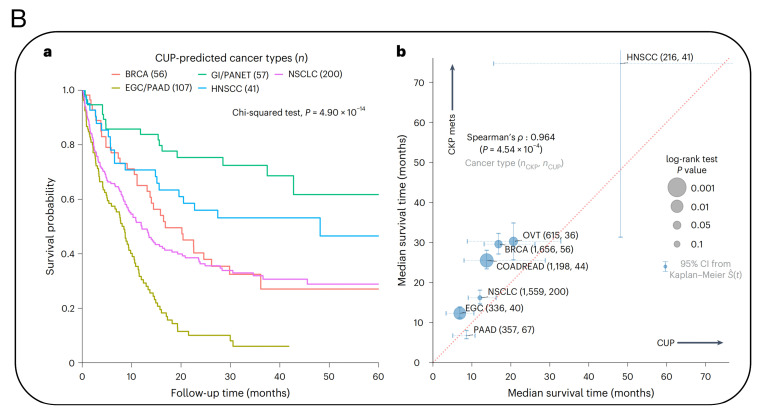
(**A**) Overview of model development and analysis workflow. (**a**) OncoNPC is an XGBoost-based classifier that was trained and evaluated using 36,465 CUP tumor samples across 22 cancer types collected from three different cancer centers. (**b**) Its performance was evaluated on the held-out tumor samples (*n* = 7289). (**c**) OncoNPC was applied to 971 CUP tumor samples at a single institution to predict primary cancer types. The association of CUP subgroups with elevated germline risk (**d**), actionable molecular alterations (**e**), overall survival (**f**) and prognostic somatic features (**g**) was investigated. (**h**) Treatment-specific outcomes were evaluated for a subset of CUP patients with detailed treatment data. (**B**) OncoNPC-based risk stratification among patients with CUP and median survival comparison between CUP and classical K. pneumoniae (CKP) metastatic cases. (**a**) Survival stratification for patients with CUP based on their OncoNPC-predicted cancer types. (**b**) Median survival was compared between patients with CUP and patients with CKP metastatic cancer [50].

Additionally, researchers from the Massachusetts Institute of Technology (MIT) and the Dana-Farber cancer institute published a paper in the prestigious medical journal *Nature Medicine*, titled “Machine learning for genetics-based classification and treatment response prediction in cancer of unknown primary” [50] (Figure 6). This study developed an AI model, OncoNPC, which analyzes DNA sequences of approximately 400 key genes influencing cancer development and uses this information to predict the origin of these “mysterious cancers”. With this AI model, they accurately classified at least 40% of tumors of unknown origin in a dataset of about 900 patients, with an average accuracy rate of 80% and an accuracy rate of up to 95% for highly credible tumor predictions (accounting for about 65% of the total). More importantly, OncoNPC can also guide the formulation of treatment plans for patients with CUP. Among CUP patients who received targeted treatment, those whose cancer types matched the model’s predictions showed better treatment outcomes. OncoNPC has increased the number of cancer patients who can receive genome-guided targeted treatments by 2.2-times. Therefore, this AI model may be used in the future to guide physicians in providing personalized treatment for patients with cancers of unknown primary.

### 2.3. In Situ Targeting Self-Assembling Probe Technology

**Figure 7:**
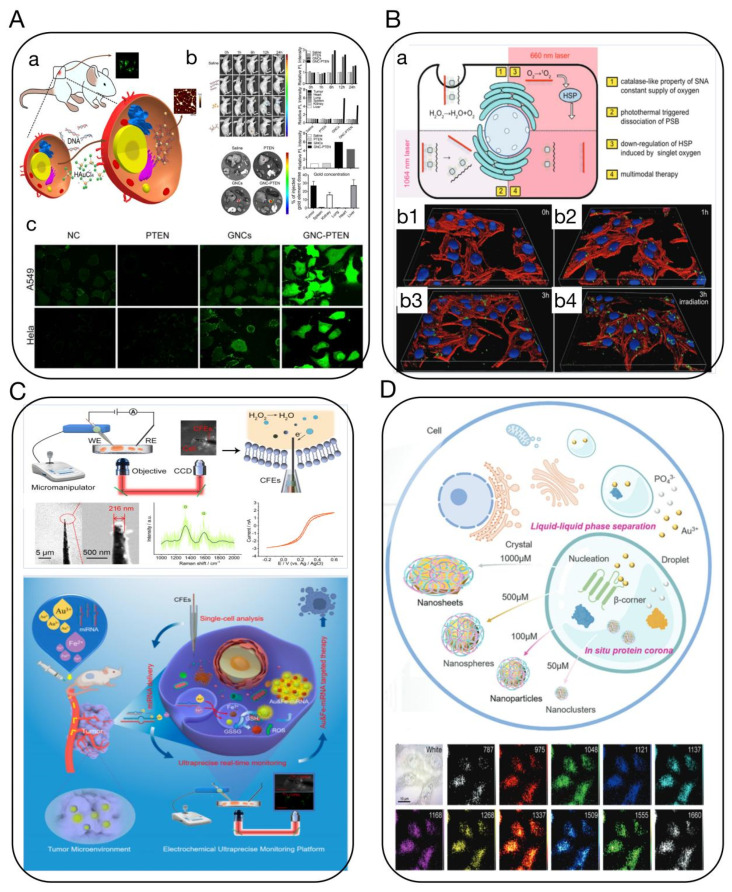
(**A**) In situ self-assembling Au-DNA complexes for targeted cancer bioimaging and inhibition [57]. (**a**) Schematic diagram of in situ self-assembled biosynthetic GNC-DNA. (**b**) Bioimaging of tumors in vivo using self-assembled biosynthesized GNC-PTEN complexes. (**c**) The ROS of different treatment groups. Fluorescent intensity indicates the strength of ROS in A549 and HeLa cells. (**B**) Tailoring photothermally triggered phase transition of multimodal cascade theranostics platform by spherical nucleic acids [58]. (**a**) Illustration of the photothermally triggered photodynamic therapy. (**b1**–**b4**) Fluorescence images of HepG2 cells incubated with 30 μg mL^−1^ of FAM-PSB for 3 h with or without NIR irradiation. (**C**) Ultraprecise real-time monitoring of single cells in tumors in response to metal ion-mediated RNA delivery [54]. (**D**) Intracellular liquid–liquid phase separation induces tunable anisotropic nanocrystal growth for multidimensional analysis [55].

Tumors often undergo a series of abnormal processes, such as hypoxia, low pH, increased oxidative stress, high glutathione (GSH), and high enzyme expression, limiting diagnostic and therapeutic approaches. However, the unique characteristics of the tumor microenvironment (TME) present possibilities for creating biological nanoprobes, ingeniously using the abnormal characteristics of tumor tissues compared to normal tissues to design and construct highly specific nanoprobes, theoretically achieving precise diagnosis of CUP [51]. Current nanoprobe technologies targeting the TME still face several challenges, including issues with the nanoparticle’s permeability, biodegradability, and biosafety [52]. Consequently, in situ self-assembling biological nanotargeting technology based on TME response is increasingly becoming a focus in cancer research [53]. Our research group has discovered that various metal ions and biochemical factors, such as target genes, can specifically interact with radicals and specific bioactive substances in tumor tissues/cells or exosomes [54] (Figure 7C). We proposed and established in vivo in situ biosynthesis and self-assembly of multifunctional biological nanoprobes, which can precisely target and accurately mark tumor sites and microlesions [55] (Figure 7D). Based on in vivo in situ self-assembling targeting technology, this approach is simpler, faster, has higher resolution, and better permeability than other nanoprobe targeting technologies, and it can achieve dynamic tracing and precise intervention of tumors and other diseases through coupling with light-, electric-, magnetic-, and force-field effects [54,56]. Therefore, in situ targeting self-assembling probe technology holds promise for multimodal, highly sensitive diagnosis of CUP [57,58] (Figure 7A,B). Furthermore, using biomimetic molecular recognition and assembly, along with surface-enhanced Raman spectroscopy, fluorescence imaging, and other techniques, we achieved accurate, high-resolution, real-time, dynamic, and rapid imaging analysis of CUP cells, exosomes, ctDNA, characteristic bioactive substances, and live lesion sites.

## 3. Targeted Treatment Strategies for CUP

**Figure 8:**
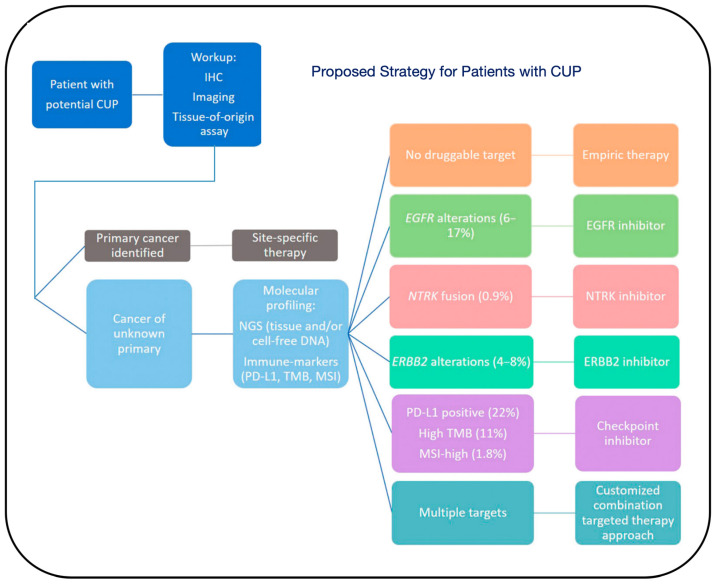
Proposed strategy for patients with CUP. Patients with potential CUP should undergo a standard workup, including immunohistochemistry (IHC), imaging, and tissue-of-origin assay, to obtain a primary cancer diagnosis. If a primary cancer is identified, patients should receive site-specific therapy. Once a patient is diagnosed with CUP, we recommend obtaining molecular profiling, including next-generation sequencing (NGS) from tissue and/or cell-free DNA, and immune-profiling, including PD-L1, tumor mutational burden (TMB), and microsatellite instability (MSI) testing, to identify actionable targets. If there is no druggable target, the patient may receive empiric therapy. However, if there are potentially targetable alterations, a targeted therapy approach based on the underlying molecular features may be considered. The percentages indicate the frequency of cognate targets among CUP patients [60].

Given the poor prognosis and resistance to treatment in most cases of CUP, precision treatment for CUP has been a pressing challenge [59]. Research on CUP largely focused on identifying the histological type of the patient’s cancer to select potentially sensitive treatment options. However, recent advances in cancer genomics and the application of targeted therapies suggest that treatment for CUP can be designed based on its molecular characteristics [13,27,60,61,62]. The continuous development of molecular detection technologies and the ongoing revelation of characteristic mutation spectra in CUP have made molecular targeted therapies, bio-nanomedicine, and immunotherapies mainstays in anti-tumor treatment, making efficient treatment of CUP possible [60] (Figure 8).

### 3.1. Molecular Targeted Therapy

**Figure 9:**
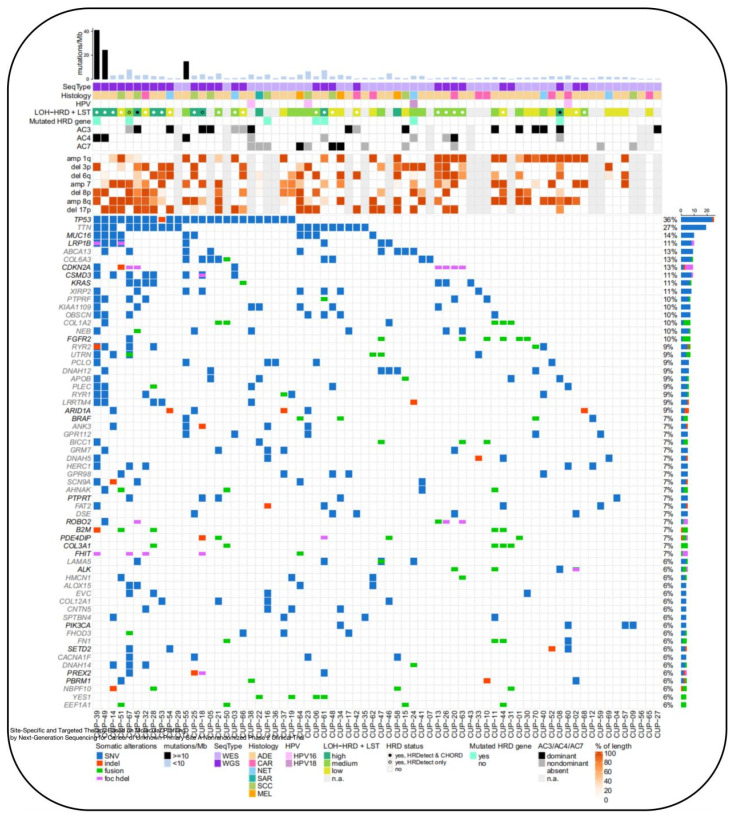
Molecular alteration landscape and TMB of CUP patient cohort. For each patient sample (x-axis), complex characteristics are provided. The bar plot on top displays the sum of non-silent somatic single-nucleotide variants (SNVs) and coding small insertions/deletions (indels) in exonic sequences per 1 Mb of the coding sequence of the genome [65].

With the advancements in genomics, the trend in CUP research is to use next-generation sequencing (NGS) to determine the molecular characteristics of the tumor and formulate personalized treatment plans, rather than relying solely on tissue type [61,63]. However, the clinical value of using genetic testing technologies to guide treatment decisions for CUP remains to be determined [63,64]. Past research on CUP was more focused on clarifying the patient’s histological type to choose potentially sensitive treatment options. Previous research suggests that treatment for cancers of unknown primary (CUP) can be based on molecular features, due to advances in understanding the genetic characteristics of cancer and the use of targeted therapies. Genetic testing in the diagnosis of CUP serves two main purposes: first, to use gene expression profiling (GEP) and molecular typing to determine the tissue origin of CUP, thereby guiding clinicians in developing appropriate treatment plans for patients; second, to utilize NGS for detecting and analyzing gene mutations related to targeted therapies, providing robust support for individualized cancer treatment. Beyond organ-specific therapy, molecular targeted therapy based on NGS is another strategy that should be considered in future experimental designs for CUP [59,65]. Currently, only a few targeted drugs are suitable for monotherapy in CUP, and combination chemotherapy continues to play a significant role in the treatment of many malignant tumors. NGS aids in predicting the primary site of CUP and guiding treatment, and this targeted approach for CUP patients has entered clinical trial phases [39]. A phase II trial assessed the impact of organ-specific and targeted therapies based on NGS, where the median overall survival was better than that of patients in the treatment-resistant group [66,67]. KRAS and other oncogenes have emerged as a novel therapeutic target, potentially providing new molecular targeted treatment options for patients diagnosed with CUP [65] (Figure 9).

### 3.2. Immunotherapy

**Figure 10:**
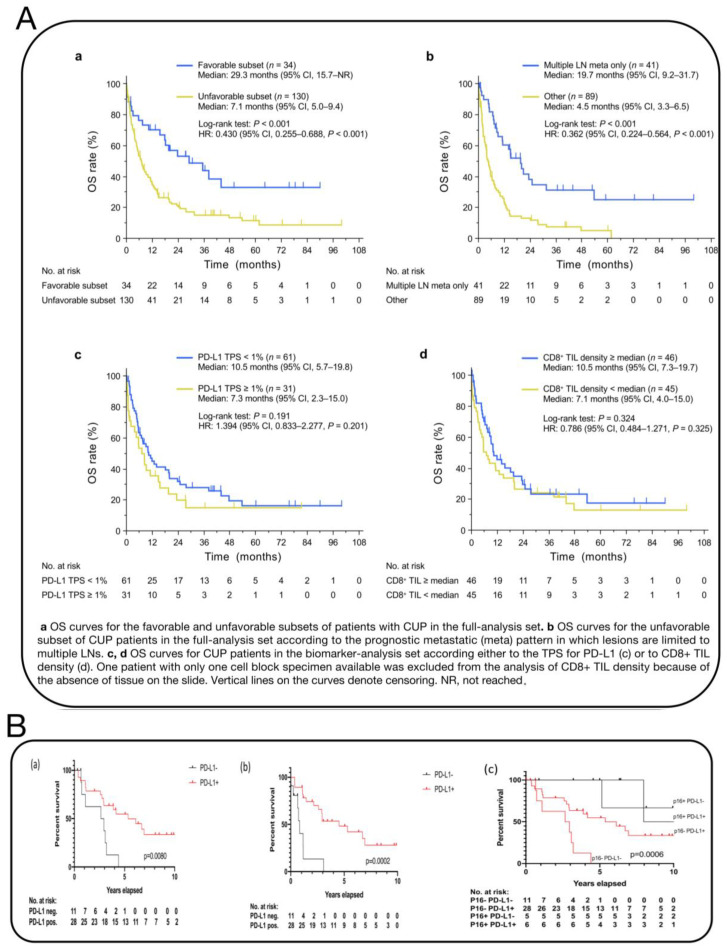
(**A**) Kaplan–Meier curves for OS [71]. (**B**) Kaplan–Meier survival analysis of overall and progression-free survival of p16-negative CUP patients in relation to PD-L1 expression. (**a**) overall survival of p16-negative CUP patients in relation to PD-L1 expression. (**b**) progression-free survival of p16-negative CUP patients in relation to PD-L1 expression. (**c**) comparison of Overall Survival of CUP patients stratifed by p16 and PD-L1 status [70].

CUP are highly aggressive and differ from other tumor types [1]. The effectiveness of immunotherapy largely depends on the tumor microenvironment (TME), but data on the immune microenvironment in CUP are scarce [68,69,70]. A study using the cBioPortal database compared the tumor cell genomes of CUP patients with those of patients with tumors suitable for ICI treatment (cervical, hepatocellular, gastric, etc.), focusing on genes related to immune checkpoint inhibition (ICI) response and resistance [71]. The genomic changes primarily involved mutations associated with ICI resistance, especially in oncogenic signaling pathways, including KRAS, STK11, and EGFR (24.7%, 10.9%, and 4.2%, respectively) [72]. Compared to other tumors eligible for ICI, CUP had a higher incidence of KRAS and STK11 changes. Transcriptome analysis of 71 cases confirmed the association between programmed death-ligand 1 (PD-L1) expression and tumor-infiltrating lymphocyte density in CUP, validating the potential benefits of immunotherapy [71] (Figure 10A). Additionally, Benedikt et al. compared PD-L1 expression in head and neck CUP squamous cell carcinoma with oropharyngeal squamous cell carcinoma, finding significantly higher PD-L1 expression in CUP. In p16-negative patients, high PD-L1 expression was found to be an independent prognostic factor. One study found that 22% of 362 patients showed tumor PD-L1 expression, and 12% exhibited high tumor mutational burden (TMB-H), offering a new option for treating CUP patients with ICIs [70] (Figure 10B). For patients with poor-prognosis CUP, the effectiveness of immunotherapy and the selection of predictive biomarkers still require more evidence-based medical research.

### 3.3. Self-Assembling Biological Nanotechnology

**Figure 11:**
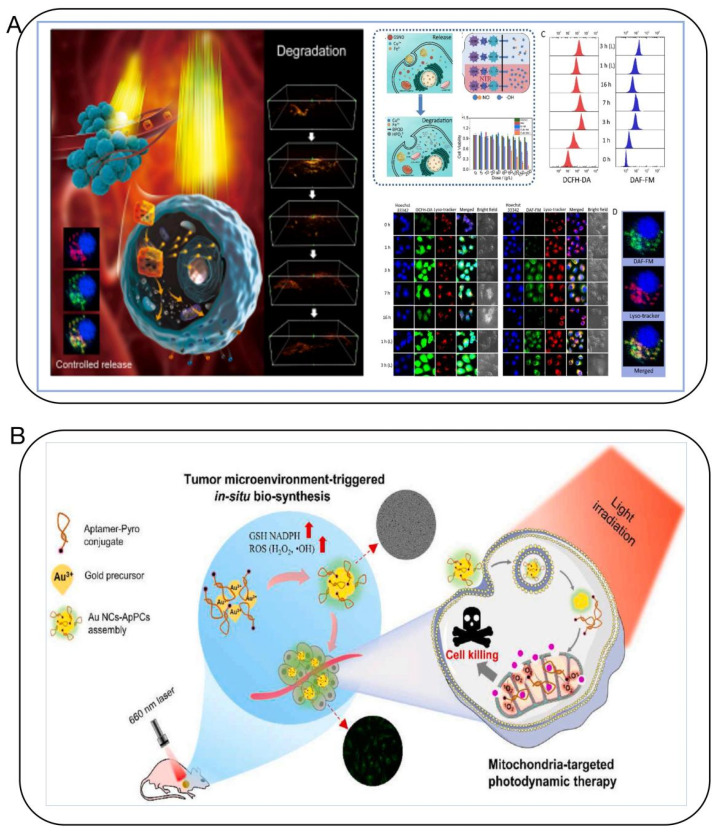
(**A**) The “Framework Exchange”-strategy-based MOF platform for biodegradable multimodal therapy [56]. (**B**) Bio-assembled specific Au NC–aptamer–pyro conjugates nanoprobe for tumor imaging and mitochondria-targeted photodynamic therapy [75].

Nanomedicine has so far been limited to ex situ self-assembly, which is hindered by poor deep-tumor penetration and blood circulation. On the other hand, in situ self-assembly-based cancer treatments have several advantages, such as improved blood circulation of monomers and long-term drug release. They have favorable delivery pharmacokinetics, low drug resistance, and the ability to target deep tumors and organelles. This can result in disruption-mediated apoptosis and enable the imaging of cellular activity for effective CUP therapy and diagnosis. The complex and heterogeneous microenvironment of tumors makes it challenging to detect early micrometastases at the macroscale. However, significant changes are already occurring at the microscopic level in biochemical factors, such as pH, hardness, intracellular nucleic acids, proteins, reactive oxygen species (ROS), reactive nitrogen species (RNS), glutathione (GSH), and other active molecules or related disease biomarkers [54,57,58,73,74,75]. Our research group combines in situ self-assembly of relevant precious metal ions with tumor-related target genes like PTEN for precision diagnostics and therapeutics [58]. We discovered that biocompatible metal ions such as gold salts can self-assemble in situ within tumor tissues/cells to form biologically responsive fluorescent gold cluster–PTEN complexes, used for long-term real-time targeted imaging and inhibiting or eliminating tumor growth and metastasis, with minimal side effects and biotoxicity, typically associated with traditional DNA transfection. These novel gene in situ self-assembling probes offer an effective, accurate cross-scale tumor targeting dynamic tracing imaging and multimodal comprehensive treatment technology and method. Our team also precisely constructed “customized” precursor compounds for in situ multimodal self-imaging-guided synchronous gas therapy and comprehensive tumor treatments like photodynamic and photothermal therapy, achieving long-term high-sensitivity imaging with fluorescence and photoacoustics and combined photothermal, photodynamic, and gas therapy for comprehensive tumor treatment and multimodal precision intervention [56] (Figure 11A). Additionally, we established a new anti-tumor strategy using in situ biosynthesized Au NCs combined with mitochondria-targeting tumor imaging and enhanced cancer PDT [75] (Figure 11B). What’s more, depending on the probe’s configuration, self-assembly can trigger cancer cell apoptosis and be used for combinatorial therapies, such as photodynamic therapy, photothermal therapy, and sonodynamic therapy, as well as imaging-guided diagnosis and therapy [76]. These approaches offer a safe, promising strategy for the effective treatment of CUP with precision diagnostics and lesion eradication.

## 4. Challenges and Future Perspectives

With advances in imaging, histopathology, and molecular diagnostic technologies, an integrated and complementary approach of various testing methods is an essential part of driving the continued advancement of the clinical diagnosis and treatment of cancer. This will aid in more accurately identifying the primary site for many patients, thereby achieving precise diagnosis and effective treatment of CUP. Recent research progress has begun to unravel the mystery of CUP, yet the enigma of cancers with unknown primary sites persists. It remains premature to guide the optimal treatment of CUP based solely on partial molecular profiling results. With the increasing application of multi-omics studies and bio-nanotargeting technology in the field of cancer, future developments will focus on more precise tracing methods for the primary lesion in CUP to better guide clinical treatment. Additionally, during research processes, there is a need for convincing methods or technology validation using animal and artificial models. In this regard, patient-derived xenografts (PDXs) involve the transplantation of tissue from patients into animals to create tumor models. Since these models directly originate from patients, they preserve histopathological characteristics and cellular heterogeneity, offering significant value in exploring and validating diagnostic and therapeutic strategies and studying rare cancer types. Moreover, organoids, derived from primary tissues or stem cells and cultivated ex vivo into self-renewing 3D models exhibiting organ functions, maintain stable phenotypes and genetic characteristics. They realistically simulate various aspects of tumors in vivo, providing distinct advantages in drug screening and applications with CRISPR technology, and they are poised for significant future applications.

Artificial intelligence technology is one of the fastest-growing fields in this era, with limitless future applications and influence. However, current AI-assisted cancer origin prediction based on whole-slide images still requires standardization and improvements in the diagnostic process. This method necessitates further training of this histology-based AI model with a larger number of cases and involvement in clinical trials to ascertain whether it can enhance diagnostic capabilities and patient prognosis. Additionally, the AI model needs to be expanded to include a wider range of other types of clinical imaging data, such as pathological and radiological images, to provide more comprehensive predictions using multiple data modalities. This will offer the AI model a holistic view of the tumor, enabling it not only to predict the type of tumor and patient prognosis but also potentially to forecast the best treatment options.

## Data Availability

Data are contained within the article.
